# Hamster Melatonin Receptors: Cloning and Binding Characterization of MT_1_ and Attempt to Clone MT_2_

**DOI:** 10.3390/ijms19071957

**Published:** 2018-07-04

**Authors:** Célia Gautier, Emilie Dufour, Clémence Dupré, Giulia Lizzo, Sarah Caignard, Isabelle Riest-Fery, Chantal Brasseur, Céline Legros, Philippe Delagrange, Olivier Nosjean, Valérie Simonneaux, Jean A. Boutin, Sophie-Pénélope Guenin

**Affiliations:** 1 PEX Biotechnologie Chimie & Biologie, Institut de Recherches Servier, 78290 Croissy sur Seine, France; gautiercelia@gmail.com (C.G.); emilie.robreau@outlook.com (E.D.); clemence.dupre@servier.com (C.D.); giulia.lizzo@yahoo.it (G.L.); sarah.caignard@servier.com (S.C.); fisab75@free.fr (I.R.-F.); chantal.brasseur@servier.com (C.B.); celine.legros@servier.com (C.L.); philippe.delagrange@servier.com (P.D.); olivier.nosjean@servier.com (O.N.); sophie-penelope.guenin@servier.com (S.-P.G.); 2 Institut des Neurosciences Cellulaires et Intégratives, 67084 Strasbourg, France; simonneaux@inci-cnrs.unistra.fr; 3 Institut de Recherches Internationales Servier, 92150 Suresnes, France

**Keywords:** melatonin, melatonin receptors, cloning, molecular pharmacology, chimeric construction, European hamster (*Cricetus cricetus*)

## Abstract

For many years, it was of interest to identify the sequences encoding the two melatonin receptors (MT_1_ and MT_2_) from various species. After publishing the basic molecular characterization of the human, rat, mouse, sheep, and platypus MT_1_, MT_2_, or Mel1c receptors, we began cloning the genes from other animals, such as birds, bats, and vipers. The goal was to advance the receptor crystallization, which could greatly contribute the understanding of the sequence/stability relationship. European hamster MT_1_ receptor was cloned for the first time from this gender, was expressed in stable form in cells, and its binding characterized with a sample of 19 melatonin ligands. Siberian hamster (*Phodopus sungorus*) expresses a non-functional MT_2_. We observed that unlike this hamster, the European hamster (*Cricetus cricetus*) does not have a stop codon in the MT_2_ sequence. Thus, we undertook the tedious task of cloning the MT_2_ receptor. We partially succeeded, sequencing the complete exon 2 and a fragment of exon 1 (from putative amino acids 12 to 38 and 77 to 323), after several years of efforts. In order to show that the protein parts we cloned were capable to sustain some binding capacities, we designed a chimeric MT_2_ receptor using a consensus sequence to replace the unknown amino acids, based on other small rodent MT_2_ sequences. This chimeric construct could bind melatonin in the nanomolar range. This work is meant to be the basis for attempts from other laboratories of the community to determine the complete natural sequence of the European hamster MT_2_ receptor. The present work is the first to show that, among the hamsters, if the Siberian is a natural knockout for MT_2_, the European one is not.

## 1. Introduction

Melatonin is a neurohormone produced only at night by the pineal gland of all mammals [1]. It is also present in the plant kingdom, where it seems to play an active antioxidant role [2,3]. Rhythmic melatonin expression depends on a circadian system comprising a master clock located in the suprachiasmatic nucleus (SCN). In mammals, its main role is to transmit the night/day rhythm from the central clock to the peripheral organs [4,5]. Thus, it is a key player in many physiological processes, and may have positive effects on numerous pathologies, including depression, which has led to the development of the melatonin receptor-targeting antidepressive drug agomelatine (Valdoxan^®^) [6,7]. The message of melatonin is transferred into cells essentially by two G-protein-coupled receptors, MT_1_ and MT_2_, and the molecular pharmacology of these receptors has been reported in human, rat, mice, and sheep [8,9,10]. In the lamb *pars tuberalis,* Piketty et al. show a possible circadian rhythm in the apparent number of melatonin receptors, with less receptors during the night [11]. Melatonin actions may also be mediated by two other kinds of targets: a nuclear receptor [12] that has been withdrawn after lack of reproducibility [13], a new hypothesis from Reiter’s group [14], and a cytosolic protein identified as the MT_3_ binding site [15]. 

To find agonists with therapeutic properties and determine their mechanism of action, the receptors in different species were described [16]. It might seem redundant to perform these Noah’s ark-type of characterizations for a pharmacological system, but differences between various laboratory species or humans can be a nightmare for the pharmacologist, as was found for the histamine H_3_ receptor [17] and the adenosine receptor species-dependent agonists and antagonists [18]. Furthermore, the search for structural biology data on receptors led to scrutiny of the sequence/stability relationship between receptors sharing a common function in various species. Indeed, understanding the thermostability of the melatonin receptors could help identify a more stable receptor that would be favorable to crystallization. Incidentally, our laboratory spent several years of work in attempting to purify a functional melatonin receptor, that led us to publish a limited breakthrough in this area [19,20]. This idea prompted us to clone different melatonin receptors from different species (birds, bats, platypus, see Table 1) to study their thermostability and attempt to purify them.

As mice and rats are nocturnal animals, it was also important to characterize the receptors from diurnal species, such as sheep, for which depressive models have been described. The sheep was considered by the melatonin community as a natural knockout for MT_2_. After obtaining a piece of sequence of the receptor described in a non-common sheep strain [21], we embarked on the difficult cloning of the ovine MT_2_ receptor, and described its molecular pharmacological properties [9]. Some of the earliest work on melatonin receptors was done on another animal species, the hamster. The hamster belongs to a family of rodents in the subfamily *Cricetinae*. It contains 25 species gathered in six or seven genera. These animals are crepuscular in the wild, and remain underground during the day, essentially to avoid predators. They can be found almost everywhere in the world. The genera are *Mesocricetus* (e.g., Syrian or golden hamster), *Phodopus* (e.g., Djungarian hamster), *Cricetus* (e.g., European hamster), *Cricetulus* (e.g., Chinese hamster), *Allocricetulus* (e.g., Mongolian hamster), *Cansumys* (e.g., Gansu hamster), and *Tscheskia* (e.g., Korean hamster). Neumann et al. carefully studied their genetic relationship [22]. In 1990, Weaver et al. initiated the cloning of MT receptors in Siberian hamsters. They found the MT_1_ receptor was complete and operational, but not MT_2_ [23], which contained two nonsense mutations within the coding region, preventing it from being functional. Since this seminal work, and without further validation outside the Siberian hamster and the Djungarian hamster gender, hamsters have been often considered, wrongly, as natural knockout animals for the MT_2_ receptor, despite that the family comprises several genera, and all were not checked for these mutations.

In the present work, we examined if the European hamster (*Cricetus cricetus*), a well-established hibernator [24], had the same mutations. We cloned and characterized the *Cricetus cricetus* melatonin MT_1_ receptor. For MT_2_, after finding that its sequence did not contain a stop codon, we attempted to clone it. We used numerous reverse transcriptase-polymerase chain reaction (RT-PCR) conditions, but only partially succeeded, as the full sequence was not obtained. We did construct a chimeric sequence, containing 85% of the cloned *Cricetus cricetus* MT_2_ sequence by replacing the missing nucleotides with a consensus sequence obtained from closely related animals. The corresponding protein shows some melatonin binding capacity, suggesting that the full sequence could be functional in this hamster gender.

## 2. Results

### 2.1. The European Hamster MT_1_ Gene Encodes a Functional Receptor

**Figure 1:**
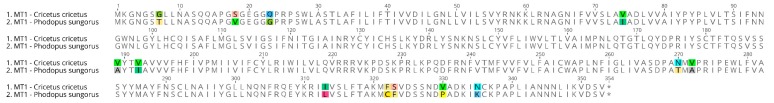
Alignment of the deduced amino acid sequences for melatonin receptor 1 from *Cricetus cricetus* and *Phodopus sungorus* (AAB17722). Protein alignment of the MT_1_ receptor from *Cricetus cricetus* and *Phodopus sungorus*. Each amino acid is depicted by its single letter symbol. Differences between the sequences are highlighted (using different colors for each amino acid).

**Figure 2:**
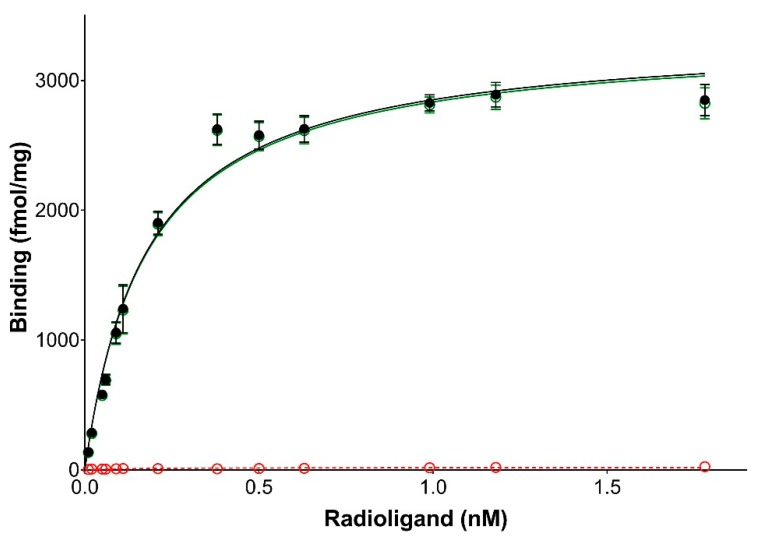
Saturation binding experiments for 2-[^125^I]-iodomelatonin. Membranes from CHO-Flp-In cells transfected with the *Cricetus cricetus* MT_1_ receptor were used to measure the binding at MT_1_ receptors. The red line represents non-specific binding, black line represents total binding, and green line represents specific binding. The expression level of the *Cricetus cricetus* MT_1_ receptor stably expressed in CHO cells was 3406 fmol/mg of protein, and the K_D_ was 127 pM.

We successfully amplified the complete coding sequence of the European hamster MT_1_ receptor. The deduced amino acid sequence was 96% identical with that of the *Phodopus sungorus* (GenBank accession number: AAB17722) melatonin receptor 1 (Figure 1). The coding sequence was submitted to the GenBank database (MG598322). The *Cricetus cricetus* MT_1_ sequence is similar to that of melatonin receptors described in other vertebrates. Next, we prepared membranes from cellular material and attempted to measure the binding of 2[^125^I]-iodomelatonin. Saturation was achieved and there was specific binding. The saturation curve (Figure 2) was similar to that obtained under similar conditions for the melatonin receptors that have been previously cloned, expressed, and characterized in our laboratory. The expression level of the *Cricetus cricetus* MT_1_ receptor from stably expressed CHO cells was 3406 fmol/mg of protein. This level is greater than what we previously found for melatonin receptors (MT_1_ and MT_2_), with expression levels ranging from 80 to 2650 fmol/mg of protein in rats and humans [8]. The dissociation constant (K_D_) was 127 pM, in line with data obtained from other MT_1_ receptors from rodents, sheep, and humans [8,9,23,25].

**Figure 3:**
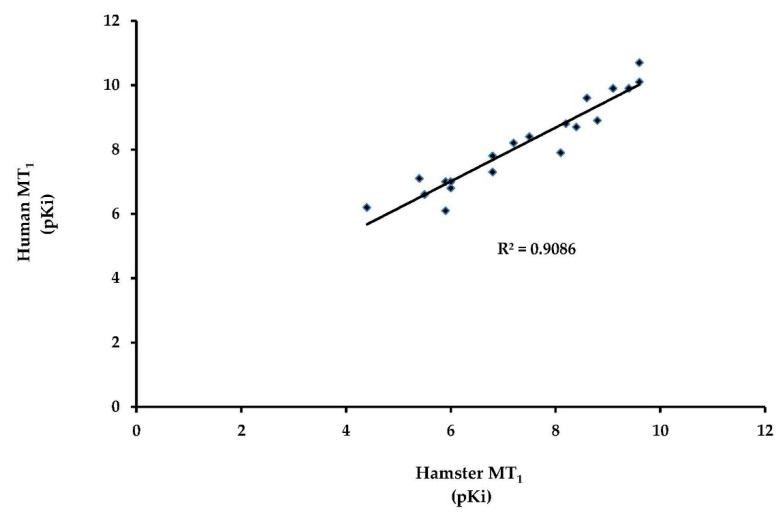
Correlation between the data obtained from hamster MT_1_ and those obtained with human MT_1_. Data were obtained in a binding assay with 2-[^125^I]-iodomelatonin as the radioligand. The hamster MT_1_ data correlate highly (*R*^2^ = 0.91) with the human MT_1_.

Membranes from untransfected CHO cells (derived from Chinese hamster (*Cricetulus griseus*) ovaries) do not sustain melatonin binding. We thus can focus on the stable expression of the *Cricetus cricetus* MT_1_ receptor in CHO cells. This receptor was expressed at workable levels, and a pharmacological profile for a series of 19 ligands was performed and compared with the human melatonin receptors MT_1_ and MT_2_ (Table 2). No major differences were recorded. Furthermore, when the data from human are simply plotted versus those from hamster, the linear correlation coefficient (*r*^2^) was 0.906, strongly suggesting that all the compounds behaved similarly in both species. There was no major difference when those data were compared with other molecular pharmacology profiles reported for MT_1_ (see Table 2 and Figure 3).

### 2.2. Cloning of Exon 1 of the Phodopus sungorus MT_2_ Gene

**Figure 4:**
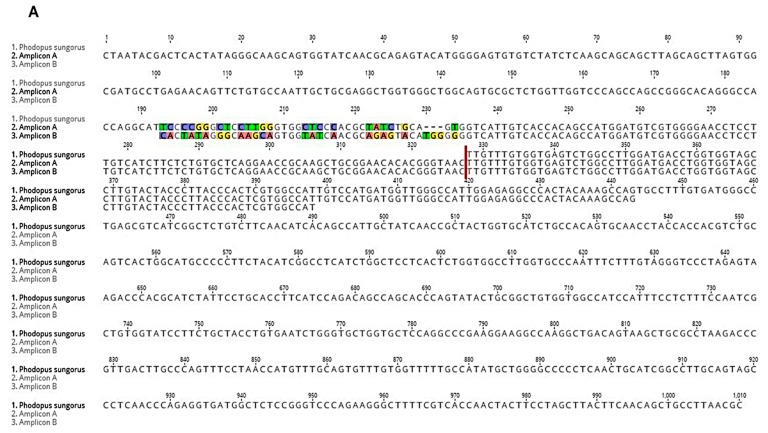

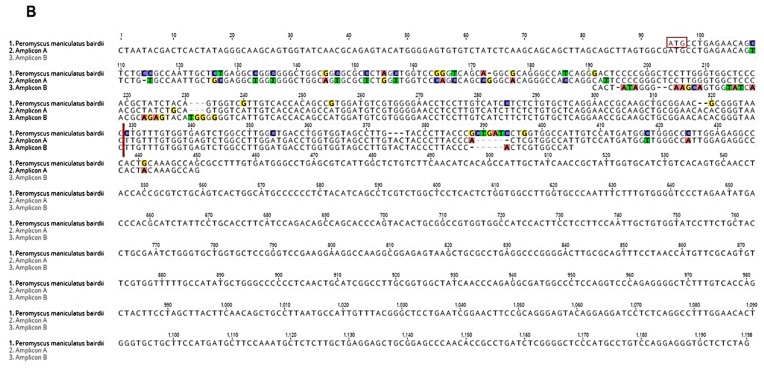
Alignment of the DNA sequences of the two amplicons from 5′ RACE with the MT_2_ sequences from *Phodopus sungorus* (**A**) or *Peromyscus maniculatus bairdii* (**B**). Two different amplicons were obtained using 5′ RACE. Exon 2 of both amplicons are identical to the *Phodopus sungorus* published sequence (**A**). The *Peromyscus maniculatus bairdii* sequence is closer to amplicon A than B (**B**). Each base is depicted by its single letter symbol. Differences between sequences are highlighted. The vertical red line represents the exon 2 position of the *Phodopus sungorus* exon 2 sequence, and the first triplet of the *Peromyscus maniculatus bairdii* MT_2_ sequence is boxed in red.

Melatonin receptors contain two exons separated by a long intron (~30,000 bp). The first exon encodes transmembrane domain I, while exon 2 encodes the other six transmembrane domains. Some amino acids of these transmembrane domains are very conserved in all species. Weaver et al. cloned an incomplete fragment of the melatonin receptor 2, corresponding to exon 2, and showed that the *Phodopus sungorus* MT_2_ melatonin receptor does not encode a functional protein, due to two nonsense mutations in the coding sequence [23]. In this species, the protein is truncated just after the transmembrane domain IV. Two different amplicons (A and B) were isolated from the *Phodopus sungorus* retina using 5′ RACE (Figure 4), and were confirmed by PCR.

The exon 2 DNA sequence of both amplicons are identical to the *Phodopus sungorus* published sequence (Figure 4A). In the sequence 5′ of exon 2, 324 bases and 135 bases were amplified for the amplicons A and B, respectively. Differences between the amplicons were observed in the beginning of amplicon B. The *Peromyscus maniculatus bairdii* MT_2_ sequence has fewer differences when compared with amplicon A than with amplicon B (Figure 4B). Interestingly, two additional bases were found at position 318 in both amplicons compared with the *Peromyscus maniculatus bairdii* MT_2_ sequence.

**Figure 5:**
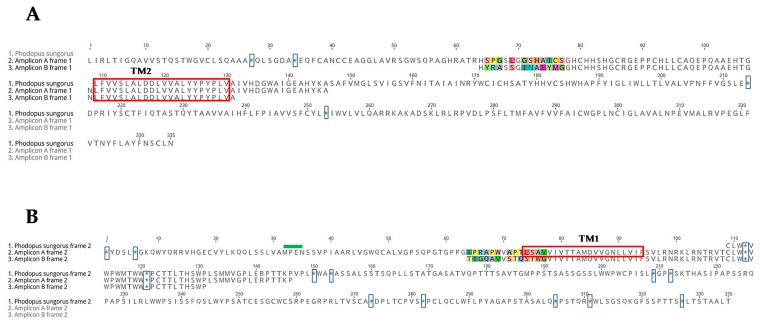
Alignment of the deduced amino acid sequences of amplicons A and B and the MT_2_ receptor of *Phodopus sungorus*. (**A**) The deduced amino acid sequences (first frame) of both amplicon A and B aligned with *Phodopus sungorus*. (**B**) Another frame is represented. Differences between the sequences are highlighted (using different colors for each amino acid). Transmembrane domains are boxed in red, and the stop codons in blue. The MPEN motif is represented with a green bar. Each base is depicted by its single letter symbol.

The deduced amino acid sequences of both amplicons were not as expected (Figure 5). The translation frame corresponding to the expected exon 2 amino acid sequence did not identify the transmembrane domain I sequence in the amplicons. In amplicon A, there was no methionine found after a stop codon in the sequence 5′ of exon 2 (Figure 5A). Using another translation frame we identified, in amplicon A, a possible transmembrane domain I sequence, as well as a methionine with an upstream stop codon (Figure 5B), but with a premature stop codon found a few amino acids after transmembrane domain I. It appears that we did not amplify the full-length MT_2_ sequence, but rather, amplicons with a conserved intronic sequence. If we delete two extra bases in the sequence of the amplicon A (in position 318 of Figure 4B) near the putative intron, the deduced amino acid sequence was closer to what was expected based for the known family of sequences of melatonin receptors 2.

### 2.3. The Cricetus cricetus MT_2_ Receptor Seems to Be Functional

#### 2.3.1. Cloning of Exon 2 of the *Cricetus cricetus* MT_2_ Receptor

**Figure 6:**
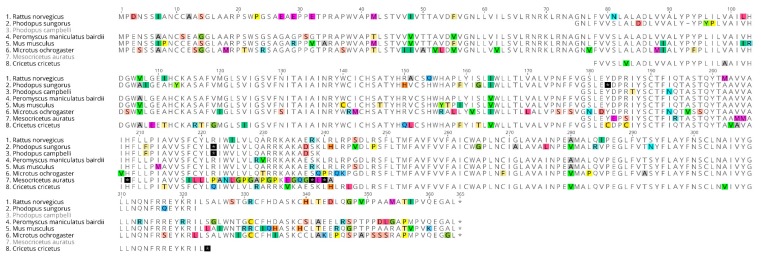
Comparison of the deduced amino acid sequences of different rodent species. Sequences for a range of rodents were downloaded from NCBI (National Center for Biotechnology Information): *Rattus norvegicus* (NM_001100641.1), *Mus musculus* (AB377276.1), *Peromyscus maniculatus bairdii* (XM_006990545.1), *Phodopus sungorus* (U57555.1), *Phodopus campbelli* (U57556.1*), Mesocricetus auratus* (AY145849.1), and *Microtus ochrogaster* (XM_005347416.1). Stop codons are highlighted in black. Each amino acid is depicted by its single letter symbol. Differences between sequences are highlighted (using different colors for each amino acid).

We successfully amplified exon 2 until the poly adenylation tail of the *Cricetus cricetus* MT_2_ gene. Surprisingly, the *Cricetus cricetus* melatonin receptor MT_2_ is not a natural knockout. Indeed, no premature nonsense mutation was observed in the deduced amino acid sequence. This receptor has seven transmembrane domains like the human, rat, or mouse receptors, but it is shorter (40 amino acids less) than the *Rattus rattus*, *Mus musculus*, and *Peromyscus maniculatus bairdii* melatonin receptor 1B (MT_2_) sequences (Figure 6).

#### 2.3.2. Cloning of Exon 1 of the *Cricetus cricetus* MT_2_ Receptor

**Figure 7:**
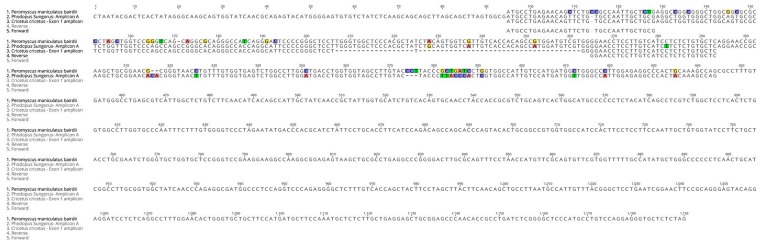
Alignment of exon 1 of the *Cricetus cricetus* cloned sequence with the melatonin receptor 2 sequences from *Peromyscus maniculatus bairdii* and *Phodopus sungorus* (*amplicon A*). Each amino acid is depicted by its single letter symbol. Differences between sequences are highlighted (using different colors for each amino acid).

We were able to isolate an incomplete region of exon 1 of the *Cricetus cricetus* melatonin receptor using a couple of primers (F0, F3, and R198) located around the presumed start methionine and the region before the predicted intron position. We amplified and sequenced an amplicon of 143 bp, instead of the expected 198 bp, a sequence 55 bp shorter than the *Peromyscus maniculatus bairdii* and *Phodopus sungorus* sequences. The sequence of our amplicon has an additional base (C) at position 171 (Figure 7) compared with the *Peromyscus maniculatus bairdii* sequence. Our exon 1 sequence is 75.5% similar to that of *Peromyscus maniculatus bairdii*, 69.4% similar to the *Mus musculus* sequence, and 68.7% similar to the *Rattus rattus* nucleic acid sequence, suggesting close similarity with the other rodent sequences.

**Figure 8:**
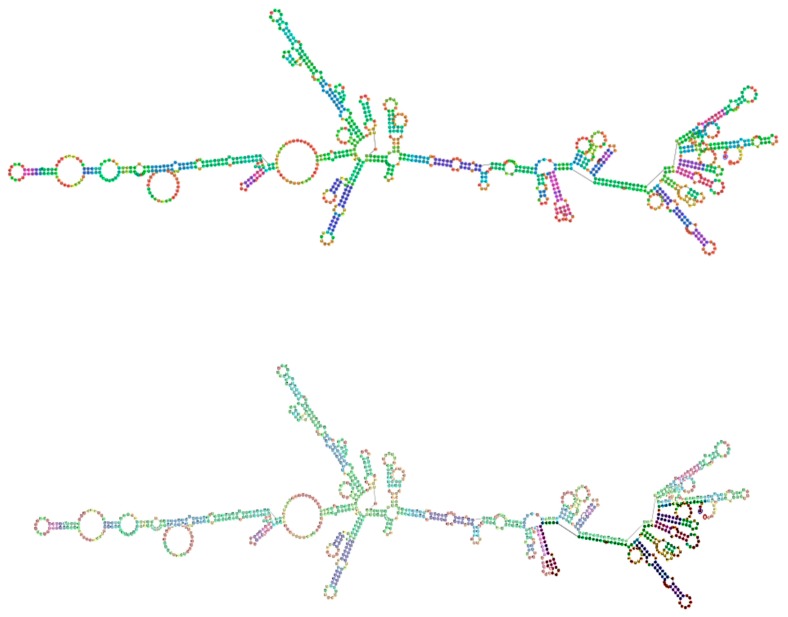
The *Peromyscus maniculatus bairdii* melatonin receptor 1B (MT_2_) structure: whole sequence (**Top**); exon 1 (**Bottom**). Geneious Pro 5.6.7. The 2D DNA structure of *Peromyscus maniculatus bairdii* melatonin receptor 1B exon 1 and the whole receptor sequence show a plethora of hairpins that may explain the difficulty in obtaining the complete sequence of our exon 1 amplicon.

In spite of the utilization of different forward and reverse primers covering both exon 1 and exon 2, we were unable to amplify a complete exon 1 sequence. The difference in size observed could be due to a technical aspect, like a stem loop. This loop could induce the polymerase to dissociate from the template. The 2D DNA structure of exon 1 of the *Peromyscus maniculatus bairdii* melatonin receptor 1B (MT_2_) and the whole receptor sequence show many hairpins in this region, which could support this stem loop hypothesis (Figure 8).

**Figure 9:**
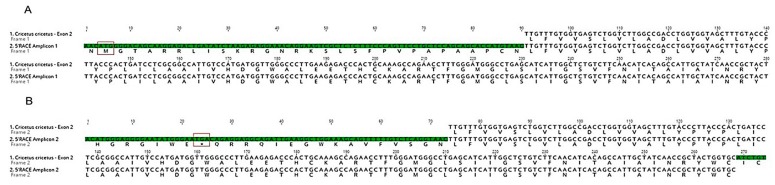
Examples of sequences obtained using 5′ RACE to obtain the *Cricetus cricetus* exon 1 sequence of the melatonin receptor 2. Nucleotides in green are not similar. The deduced amino acid of the amplicon 1 shows a methionine 5′ of exon 2 (squared in red), but the pattern for transmembrane domain I was not observed, while amplicon 2 shows a stop codon (boxed in red) 5′ of exon 2, but no methionine.

Different amplicons were isolated from 5′ RACE experiments; two examples are presented in Figure 9A,B. The sequence of exon 2 was as expected. None of the different 5′ regions amplified were as expected. Some amplicons, like amplicon 1 (Figure 9A), have a possible start methionine but no transmembrane domain. We also observed some amplicons with a stop codon in the 5′ region, but without a start methionine (Figure 9B).

Interestingly, two other amplicons that were sequenced contained the expected exon 2 region, but with an upstream sequence homologous to the *Cricetulus griseus* melatonin receptor 1B (MT_2_) pseudogene (NG_051276.1). Furthermore, at least some intronic regions seem to be conserved in these amplicons. We tried to amplify exon 1 from the MT_2_ receptor with primers designed from non-coding regions using genomic DNA as a template. Several PCR products were amplified, and all were subcloned, but none of the sequences obtained were specific. The same result was observed for PCR reactions performed with primers based on the coding region of the *Cricetulus griseus* sequences.

#### 2.3.3. Molecular Pharmacology

**Figure 10:**
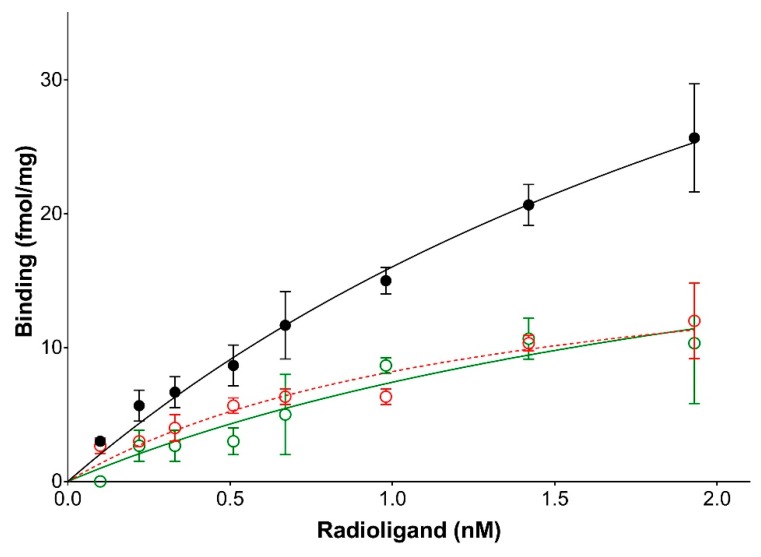
Saturation binding experiments for 2-[^125^I]-iodomelatonin. Whole COS7 cells (50,000) transfected with the *Cricetus cricetus* chimeric MT_2_ receptor were used to measure the binding at chimeric MT_2_ receptors. The red line represents non-specific binding, black line represents total binding, and green line specific binding.

Next, we attempted to measure the binding of 2-[^125^I]-iodomelatonin on whole COS7 cells transfected with *Cricetus cricetus* chimeric MT_2_. Saturation was obtained and there was poor specific binding (as per melatonin receptor standards). The saturation curve (Figure 10) was not similar to those obtained under similar conditions for the melatonin receptors that have been cloned, expressed, and characterized in our laboratory. Although saturation conditions were not obtained, a linearization of the Scatchard plot was determined, and gave a Bmax of 47.57 fmol/mg protein and a K_D_ value of 5 nM. The expression was low, compared with the other MT_2_ receptors from different species. The K_D_ value compares poorly with previous data from other species, including 0.041 nM for ovine MT_2_ [9], 0.08 nM for human MT_2_ [25], 0.022 nM for mouse MT_2_ [10], and 0.13 nM for rat MT_2_ [8]. This variability is essentially due to the nature of the host cells (HEK, CHO).

## 3. Discussion

In mammals, melatonin is mainly involved in the transmission of circadian and seasonal information. This neurohormone acts essentially by two G-protein-coupled receptors, MT_1_ and MT_2_. Melatonin transmits photoperiodic and seasonal signals mainly through MT_1_ [26]. Weaver et al. showed that MT_2_ from the Siberian hamster was not functional due to nonsense mutations in the coding region of the receptor cDNA [23]. Here, we analyzed the functionality of both MT_1_ and MT_2_ in the European hamster. The European hamster MT_1_ receptor was cloned, overexpressed in CHO cells, and its binding capacity studied. The alignment of the *Cricetus cricetus* receptor with *Phodopus sungorus* MT_1_ showed high similarity at the amino acid level. The affinity of this receptor (127 pM) for melatonin is in the same range that we usually observe for melatonin receptors expressed similarly [27]. Technical cloning difficulties have curbed studies of melatonin receptors. For instance, for a long time, it was believed that the ovine MT_2_ gene had evolved into a pseudogene that produced no mRNA [28]. Finally, we showed the existence of a functional MT_2_ receptor in sheep [9]. We used different 5′ RACE conditions to complete the published sequence of the Siberian hamster MT_2_. Two different amplicons were cloned, and though the nucleic acid sequence was close to the *Peromyscus maniculatus bairdii* sequence, the deduced amino acids were not as expected. Interestingly, when compared with the *Peromyscus maniculatus bairdii* sequence, the amplicons cloned contain, among other changes, two extra bases 5′ of the presumed intron. These bases could induce the observed frameshift and be due to intronic conservation. Exon 2 of the *Cricetus cricetus* MT_2_ receptor was cloned, and surprisingly, our sequence does not contain a single premature codon stop. Using tissues from hibernating hamsters, an incomplete exon 1 sequence was amplified. This fragment was shorter than expected, and despite multiple trials, it was not possible to determine if the exon 1 of the *Cricetus cricetus* MT_2_ is shorter than MT_2_ from other species, or if the missing sequence was due to technical problems. The presence of a plethora of hairpins in the exon 1 structure could explain the difficulty in obtaining its complete sequence. The engineered chimeric MT_2_ receptor showed poor affinity for melatonin. This result indicates that the full length *Cricetus cricetus* MT_2_ receptor could be functional and bind melatonin. Using 5′ RACE, transcripts with intronic conservation in the sequence 5′ of exon 2 were amplified, and the deduced amino acids were not as expected. These experiments were performed using tissues from non-hibernating hamsters, and should be reproduced using tissues from hibernating hamsters to determine if these transcripts are only found in non-hibernating animals. The impact of the chimeric construct on the expression level (the lowest MT_2_ expression in our laboratory) and the melatonin affinity (5 nM, about 50 times poorer than we usually find for the MT_2_ proteins in our laboratory) is certainly important. Nevertheless, the elements we discovered, and particularly, the absence of a stop codon impairing the expression of a functional receptor, as found in most other hamster species, are important. We also believe that by introducing the artificial N-terminal sequence, we have proven that the rest of the construct is functional. More molecular biology work will be needed to decrypt the European hamster MT_2_ receptor. Our data suggest that transcripts with intronic conservation are expressed in the non-hibernating *Cricetus cricetus* and *Phodopus sungorus*. These transcripts could be mainly expressed to regulate the wild-type MT_2_ receptor. The functional MT_2_ could be expressed only during a specific physiological state, such as hibernation. We did previously show that MT_2_ is regulated during the hibernation cycle in the European hamster [29]. Regulation using pseudogene transcript expression would permit a rapid increase in the functional MT_2_ receptor when the European hamster enters hibernation. Both the entry and the end of hibernation are very fast processes (a few hours) during which major adaptations are observed concerning metabolism, body temperature, etc. These profound modifications have to be highly regulated, and pseudogene regulation facilitates a very specific and rapid gene regulation. Sano et al. [30] showed a hibernation-specific alternative splicing for the cold-inducible RNA-binding protein (CIRP) in the hearts of Syrian hamsters. They demonstrated that the full length CIRP was expressed during the hibernation state; therefore, a C-terminally truncated protein was also expressed in non-hibernating animals. This truncated form could play a dominant-negative role over the full length CIRP [30]. This kind of regulation could be of major importance and a determinant for the key genes implicated during hibernation.

In conclusion, we showed that exon 2 of the *Cricetus cricetus* MT_2_ receptor does not contain a premature stop codon. Our study results suggest that MT_2_ transcripts with intronic conservation are expressed, maybe only during particular physiological state(s), in both *Cricetus cricetus* and *Phodopus sungorus*. Finally, the premature stop codons in the *Phodopus sungorus* MT_2_ sequence could be due to a reading frame shift induced by intronic conservation.

## 4. Materials and Methods

### 4.1. Animals and Tissue Collection

#### 4.1.1. Cricetus cricetus

European hamsters (*Cricetus cricetus*) are bred in-house (Chronobiotron, CNRS-UMS 3415). Adult male hamsters were individually maintained in cages with food and water given ad libitum during the study. Animals were euthanized by CO_2_ inhalation and brains; liver or heart were rapidly removed, rinsed in cold Ringer’s solution, frozen in liquid nitrogen, and then stored at −80 °C until further use. Experimental procedures were verified by a local ethical committee and authorized by the French Direction de la Recherche et de l’Innovation (authorization number 01546.02; July 6, 2015). The induction of the hibernation state was performed as previously described [29].

#### 4.1.2. Phodopus sungorus

Male Siberian hamsters (*Phodopus sungorus*) were bred in-house (Chronobiotron, CNRS-UMS 3415). Adult male hamsters were individually maintained in cages with food and water given ad libitum during the study. Hamsters were maintained in a short photoperiod (10 h light and 14 h dark) at 20 °C ambient temperature. Animals were euthanized by CO_2_ inhalation. The brain and retina were rapidly removed, rinsed in cold Ringer’s solution, frozen in liquid nitrogen, and then stored at −80 °C until cloning.

### 4.2. RNA Extraction

Total RNA was extracted using the RNeasy^®^ Lipid Tissue Mini Kit (Qiagen, Valencia, CA, USA) according to the manufacturer’s protocol. Briefly, 100 mg of organs (if possible) were added to 1 mL QIAzol Lysis Reagent containing one 5 mm stainless steel bead (Qiagen). Tissues were disrupted and homogenized using a tissue lyser for 2 min at 20 Hz, and homogenates were incubated at room temperature (RT) for 5 min. Then, 200 µL of chloroform (Sigma, St. Louis, MO, USA) was added. After incubation for 5 min at RT, the homogenates were centrifuged at 12000*g* for 15 min at 4 °C. The upper aqueous phase containing RNA was transferred to a new tube, and then the QIAcube was used per the manufacturer’s protocol for animal tissue (Qiagen). Total RNA samples were stored at −80 °C until use.

### 4.3. Cloning of the Phodopus sungorus MT_2_ Gene

*Phodopus sungorus* retina total RNA (1 µg) samples were converted into cDNA using the SMARTer^®^ RACE (Rapid amplification of cDNA ends) 5′/3′ Kit (Takara Bio USA, Mountain View, CA, USA) according to the manufacturer’s instructions. The cDNA was used in PCR amplification reactions containing the SMART^®^ RACE kit universal primer mix (UPM) and gene specific antisense exon 2 primers (5′-GACGTGGTGGTAGGTTGCACTGT-3′) based on the *Phodopus sungorus* (U57555) sequence. The PCR reaction (50 µL) was set up with Q5 High-Fidelity DNA Polymerase (NEB, Ipswich, MA, USA) according to the manufacturer’s protocol. The PCR conditions were as follows: 5 cycles of 94 °C for 30 s, 72 °C for 3 min; 5 cycles of 94 °C for 30 s, 70 °C for 30 s, 72 °C for 3 min; and 25 cycles of 94 °C for 30 s, 68 °C for 30 s, 72 °C for 3 min. Using a dilution of the first PCR product (1/10), a second PCR was performed using the KAPA 2G Robust PCR Kit (Kapa Biosystems Wilmington, DE, USA). The reverse primer 5′-CTGGCTTTGTAGTGGGCCTCTC-3′ was used with the UPM forward primers. The last PCR reaction was performed, in the same way, using a dilution of the second PCR product and the reverse primers: 5′-CTGGCTTTGTAGTGGGCCTCTC-3′ or 5′-CATCCAAGGCCAGACTCACCACAA-3′. To confirm the results obtained by 5′ RACE, primers were designed from the sequence obtained. Forward primers were designed around the putative start methionine and reverse primers flanked the end of exon 2. *Phodopus sungorus* brain total RNA (5 µg) was converted into cDNA using the oligo dT primers and the PrimeScript™ High Fidelity RT-PCR Kit (Takara Bio USA) according to the manufacturer’s instructions. The PCR reactions (50 µL) were set up with Q5 High-Fidelity DNA Polymerase (NEB), following the manufacturer’s protocol, with the following two pairs of primers: forward ATGCCTGAGAACAGTTCTGTGCCAATTGCTGCG and reverse CCAGATTCACAGGTAGCAGAAGGATAC; and forward GCTGCGAGGCTGGTGGGCTGGCAGTGCGCT and reverse CCGTAGACAATGGCGTTAAGGCAGCT.

### 4.4. Cloning of the Cricetus cricetus MT_1_ Gene

*Cricetus cricetus* liver or heart total RNA was converted into cDNA with oligo dT primers and the PrimeScript™ High Fidelity RT-PCR Kit (Takara Bio USA) according to the manufacturer’s instructions. The primers used to amplify the MT_1_ gene were based on the *Phodopus sungorus* (U14110), *Mus musculus* (NM_008639), and *Cricetulus griseus* (XM_003507458) sequences. The primers were as described in Table 3A. The PCR reactions (50 µL) were set up with Q5 High-Fidelity DNA Polymerase (NEB) or Phusion Hot Start High Fidelity DNA Polymerase (Thermo Fisher Scientific, Waltham, MA, USA), following the manufacturer’s protocol.

### 4.5. Cloning of the Cricetus cricetus MT_2_ Gene

#### 4.5.1. Cloning of a Partial Fragment of Exon 1 of the MT_2_ Receptor from *Cricetus cricetus*

*Cricetus cricetus* hypothalamus total RNA (5 µg) from hibernating animals was converted into cDNA with the oligo dT primers and the PrimeScript™ High Fidelity RT-PCR Kit (Takara Bio USA) according to the manufacturer’s instructions. Primers to amplify the MT_2_ gene were based on the *Phodopus sungorus* (see above) and *Peromyscus maniculatus bairdii* (XM_006990545.1) sequences. The forward primer sequences were 5′-ATGCCTGAGAACAGTTCTGTGCCAATTGCTGCG-3′ (F0) and 5′-GCTGCGAGGCTGGTGGGCTGGCAGTGCGCT-3′ (F3), and the reverse sequence was 5′-GAGCACAGAGAGGATGACAAGGA-3′ (R198). Forward primers included the start codon, while the reverse primer included the transmembrane domain 1. The PCR reactions (50 µL) were set up using the KAPA HiFi HotStart PCR Kit (Kapa Biosystems Wilmington, DE, USA) and contained 2 µL cDNA, 1X KAPA HiFi Buffer GC, 0.3 mM dNTPs, 0.3 µM primers, and 0.5 U KAPA HiFi HotStart DNA Polymerase. The PCR conditions were 95 °C for 5 min; 10 cycles as follows: 98 °C for 20 s, 72 °C for 15 s, 72 °C for 1 min; for these 10 cycles the annealing temperature is from 72 °C to 62 °C (−1°/cycle) and finally, 30 cycles of 98 °C for 20 s, 65 °C for 15 s, 72 °C for 1 min; followed by 1 min at 72 °C. When a second amplification was needed, it was performed using the same conditions and primers, and a dilution (1:10) of the first PCR product was used as the template. To complete the sequencing of exon 1, we designed reverse primers before the putative intron. Primers were designed from the *Phodopus sungorus* and *Peromyscus maniculatus bairdii* (XM_006990545.1) sequences. PCR reactions were carried out with the F0 and F3 forward primers (as listed in Table 3B) using the same conditions described just above. Other reverse primers were designed in the transmembrane domain 4 and in the 3′-UTR region of transmembrane domain 7. The forward primer was designed in transmembrane domain 1. Primers for the MT_2_ gene were designed from *Phodopus sungorus* (see above) and *Peromyscus maniculatus bairdii* (XM_006990545.1) sequences as follows: forward primer TGTCGTGGGGAACCTCCTTGTCATCCTC and reverse primers AGAGTGAGGAGCCAGACGAGGGTGATG and CAAGGCAGCATTTGGAAGATTCATGGAAGCAG. The reverse primers are located in exon 2. *Cricetus cricetus* hypothalamus total RNA (5 µg) from hibernating animals was converted into cDNA using oligo dT primers and the Super Script III First Strand Synthesis System for RT PCR Kit (Thermo Fisher Scientific) according to the manufacturer’s instructions. The PCR reactions (50 µL) were set up with Q5 High-Fidelity DNA Polymerase (NEB) according to the manufacturer’s protocol.

#### 4.5.2. 5′ RACE of the *Cricetus cricetus* MT_2_ Gene

*Cricetus cricetus* brain total RNA (1 µg) from non-hibernating animals was converted into cDNA using the SMARTer RACE cDNA Amplification Kit (Takara Bio USA), according to the manufacturer’s instructions. Briefly, RNA and the 5′ CDS Primer A were incubated at 72 °C for 3 min and 42 °C for 2 min. The RT-PCR reactions (20 µL) contained 2 µL of 5× First Strand Buffer, 1 µL 20 mM DTT, 1 µL 10 mM dNTP Mix, 1 µL of the SMARTer IIA oligo, 0.25 µL RNAse Inhibitor, and 1 µL SMARTScribe Reverse Transcriptase. This mix was incubated at 42 °C for 90 min and 70 °C for 10 min. The PCR reactions (50 µL) were performed with Q5 High-Fidelity DNA Polymerase (NEB) following the manufacturer’s protocol. Two rounds were carried out using the NUP forward primer (5 µL) and the following reverse primers (2.5 µL): 5′-CAAAGAAATTGGGCACCAAGGCCACCAG-3′ for the first round and 5′-GCACCAGTAGCGGTTGATAGCAATGG-3′ for the second round. The PCR conditions were 98 °C for 3 min; 10 cycles as follows: 98 °C for 20 s, 75 °C for 30 s, 72 °C for 30 s; for these 10 cycles the annealing temperature is from 75 °C to 65 °C (−1°/cycle) and finally, 30 cycles of 98 °C for 20 s, 72 °C for 30 s; and 30 cycles of 98 °C for 10 s, 65 °C for 30 s, 72 °C for 30 s; followed by 4 min at 72 °C. For the second round, the elongation was 65 °C for 30 s. Another PCR reaction was performed using the same conditions described above, but with the following reverse primers: 5′-GCACCAGTAGCGGTTGATAGCAATGG-3′ for the first round and 5′-GATGTTGAAGACAGAGCCAATGATGC-3′ for the second round.

#### 4.5.3. Further Attempts to Clone Exon 1 of the *Cricetus cricetus* MT_2_ Receptor

**Figure 11:**
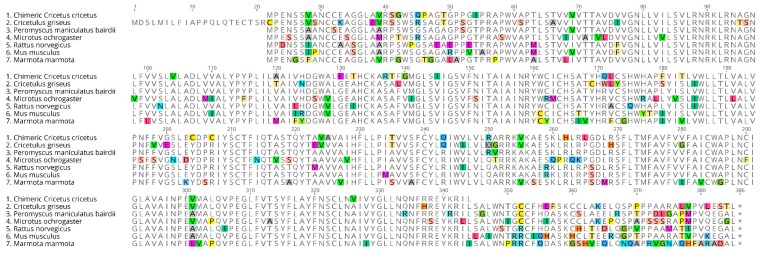
Alignment of various mammalian melatonin MT_2_ receptor sequences. Alignment of various mammalian melatonin MT_2_ receptor sequences and our chimeric receptor. Sequences for a range of rodents were downloaded from NCBI (National Center for Biotechnology Information): *Rattus norvegicus* (NM_001100641.1), *Mus musculus* (AB377276.1), *Peromyscus maniculatus bairdii* (XM_006990545.1), *Microtus ochrogaster* (XM_005347416.1), *Marmotta marmotta* (XM_015489993), and *Cricetulus griseus* (XM_007636225). Each amino acid is depicted by its single letter symbol. Differences between sequences are highlighted (using different colors for each amino acid).

One of the major challenges was to design appropriate primers that were specific and had appropriate PCR melting temperatures. We designed primers based on the Chinese hamster (*Cricetulus griseus*) MT_2_ gene sequence (XM_007636225.1), which is quite different from the other melatonin receptors. The 5′-UTR region is longer, about 22 amino acids, than the other receptors, and the start methionine is not directly followed by a PENS-like pattern, which is conserved in many species (Figure 11). An extra nucleotide before the putative start codon could induce the loss of the methionine. This difference may be explained by a genetic degeneration in the cell line sequenced or a sequencing error and these explanations were taken into account when designing the primers based on this species.

*Cricetus cricetus* retina total RNA (5 µg) from hibernating animals was converted into cDNA with oligo dT and random hexamer primers using the PrimeScript™ High Fidelity RT-PCR Kit (Takara Bio USA), according to the manufacturer’s instructions. The PCR reactions (50 µL) were set up with Q5 High-Fidelity DNA Polymerase (NEB) following the manufacturer’s protocol and using 2 µL of template cDNA. In brief, the PCR conditions were 98 °C for 3 min; 35 cycles of 98 °C for 10 s, 60 °C for 30 s, 72 °C for 30 s; followed by 2 min at 72 °C. Long forward primers were designed from the *Cricetulus griseus* MT_2_ receptor (XM_007636225.1) sequence, around the start codon (Table 3C). The reverse primer was designed based on the 3′ end coding region of the *Cricetus cricetus* receptor.

We also tried to obtain the sequence of the MT_2_ gene using genomic DNA as a template. Genomic DNA was extracted using the AllPrep^®^ DNA/RNA Mini Kit (Qiagen, Valencia, CA, USA) according to the manufacturer’s instructions. The PCR reactions (50 µL) were set up with Q5 High-Fidelity DNA Polymerase (NEB) following the manufacturer’s protocol and using 3 µL of genomic DNA. The PCR conditions were as follows: 98 °C for 3 min; 40 cycles of 98 °C for 10 s, 60 °C for 30 s, 72 °C for 30 s; followed by 2 min at 72 °C. Forward and reverse primers were designed based on non-coding regions located in the 5′-UTR (forward primers) and intron regions (reverse primer) of the *Cricetulus griseus* melatonin receptor 1B pseudogene (NG_051276.1) (Table 3D), and flanked the coding region.

Several bands were amplified and PCR products were inserted into the linear cloning plasmid, pJazz, using the BigEasy Kit (Lucigen, Middleton, WI, USA) according to the manufacturer’s instructions. This vector provides several advantages when the target DNA is difficult to clone in conventional vectors, particularly for GC-rich genes. All PCR products were purified using the High Pure Purification kit (Roche, Mannhein Germany). Eluted fragments were end repaired using the Lucigen’s DNATerminator^®^ End Repair Kit (Lucigen, Middleton, WI, USA), purified, inserted into the plasmid pJazz, and finally transformed into BigEasy TSA™ electrocompetent cells.

#### 4.5.4. Cloning of Exon 2 and the 3′-UTR of the *Cricetus cricetus* MT_2_ Receptor

The following PCR reactions were performed to obtain the exon 2 sequence of the *Cricetus cricetus* MT_2_ gene. *Cricetus cricetus* liver total RNA (1 µg) from non-hibernating animals was converted into cDNA with the oligo dT primer and the PrimeScript™ High Fidelity RT-PCR Kit (Takara Bio USA) according to the manufacturer’s instructions. The PCR reactions (50 µL) were set up with Phusion Hot Start High Fidelity DNA Polymerase (Thermo Fisher Scientific) according to the manufacturer’s protocol using 5′-TTGTTTGTGGTGAGTCTGGTCTTGG-3′ for the forward primer and 5′-GCCCATAGACAATGACGTTAAGGCAG-3′ for the reverse primer. The PCR conditions were 98 °C for 30 s; 40 cycles of 98 °C for 10 s, 65 °C for 30 s, 72 °C for 1 min; followed by 10 min at 72 °C.

*Cricetus cricetus* brain total RNA (1 µg) from non-hibernating animals was converted into cDNA with a modified oligo (dT) primer and 3′-CDS Primer A using the SMARTer RACE cDNA Amplification Kit (Takara Bio USA) according to the manufacturer’s instructions. The PCR reaction (50 µL) was set up with Q5 High-Fidelity DNA Polymerase (NEB) following the manufacturer’s protocol. For the first round, the 5′-CAGAGGTGATGGCTCTCCAGGTCCCAG-3′ primer and the UPM primer from the commercial kit were used. The PCR conditions were: 98 °C for 3 min; 40 cycles of 98 °C for 10 s, 65 °C for 30 s, 72 °C for 30 s; followed by 2 min at 72 °C. The second round was performed using this first PCR product as the template (2 µL) and the 5′-TGTCACCAGCTACTTCCTAGCTTACTTCA-3′ primer and NUP primer (2 µL) from the commercial kit.

### 4.6. Subcloning and Sequencing

Amplicons were separated on 1% agarose gels stained with ethidium bromide; the gel bands were revealed with U Genius (Syngene, Frederick, MD, USA). When several bands were observed, PCR products were purified using the High Pure Purification kit (Roche, Mannhein, Germany). Eluted DNA was inserted into the blunt pJET vector using the CloneJET PCR Cloning Kit (Thermo Fisher Scientific) and transformed into DH10β chemically competent *Escherichia coli* cells (NEB). Forward and reverse sequencing reactions were performed with the Big Dye Terminator Cycle Sequencing Ready Reaction Kit (Applied Biosystems, Life Technologies Corporation, Carlsbad, CA, USA) using the same primers used for amplification or vector primers. When needed, due to the high GC content, sequencing reactions were carried out with 10% DMSO. Sequencing products were purified using the BigDye XTerminator^®^ Purification Kit (Thermo Fisher Scientific) and run on an ABI 3730 XL automated sequencer (Applied Biosystems). Data were analyzed with Sequencher^®^ version 5.4.6 DNA sequence analysis software (Gene Codes Corporation, Ann Arbor, MI, USA) and Geneious Pro 5.6.7 (Biomatters, Auckland, New Zealand).

### 4.7. Establishment of Transient CHO-Flp-in MT_1_ Cell Lines

CHO-Flp-In cells (Thermo Fisher Scientific) were cultivated in the presence of zeocin (0.1 mg/mL). The cells were co-transfected with *Cricetus cricetus* (MG598322) MT_1_/pcDNA5-FRT (*Cricetus cricetus* MT_1_) and the Flp recombinase expression pOG44 plasmid using PEIpro (Invitrogen, Carlsbad, CA, USA). The CHO cells stably expressing *Cricetus cricetus* MT_1_ were selected using hygromycin (0.6 mg/mL). CHO-Flp-In-*Cricetus cricetus* MT_1_ cells were grown in HAM F12 with 2 mM glutamine and supplemented with 10% fetal calf serum. Cells were selected using antibiotic pressure (hygromycin) for 72 h. The resistant cells were pooled and the presence of the transgene confirmed by RT-PCR (not shown) using 500,000 cells. Then, up to 2 × 10^9^ cells were grown and used to prepare the cell membranes used below.

### 4.8. Membrane Preparations

Membranes were prepared as previously described for CHO cells expressing the sheep MT_2_ receptor [9]. Briefly, CHO cells expressing the *Cricetus cricetus* MT_1_ melatonin receptor were grown to confluence, harvested in phosphate buffer containing 2 mM EDTA, and centrifuged at 1000× *g* for 5 min at 4 °C. The resulting pellet was suspended in 5 mM Tris-HCl (pH 7.4) containing 2 mM EDTA and homogenized using a Kinematica polytron. The homogenate was then centrifuged (20,000× *g*, 30 min, 4 °C), and the resulting pellet suspended in 75 mM Tris-HCl (pH 7.4) containing 2 mM EDTA and 12.5 mM MgCl_2_. The protein content was determined according to the method of Lowry et al. [31] using the Bio-Rad kit (Bio-Rad Laboratories, Hercules, CA, USA). Aliquots of membrane preparations were stored in binding buffer (50 mM Tris-HCl, pH 7.4 containing 5 mM MgCl_2_ and 1 mM EDTA) at −80 °C until use.

### 4.9. Establishment of Transient COS7/Chimeric MT_2_ Cell Lines

#### 4.9.1. Design of the Chimeric MT_2_ Receptor

**Figure 12:**
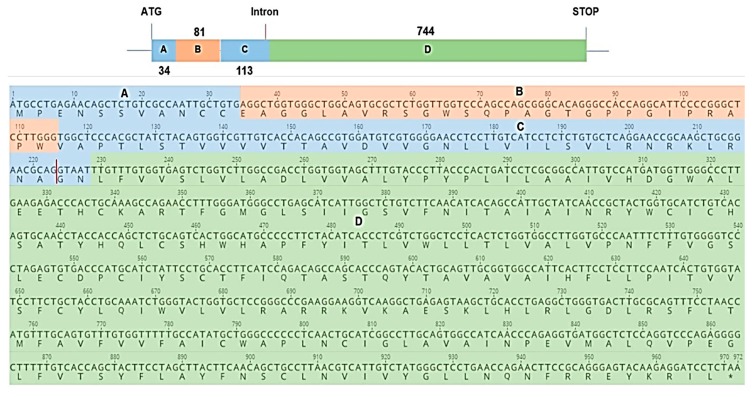
Sequence of the engineered chimeric melatonin receptor 2. Part A and C in blue are the result of a consensus of six MT_2_ mammalian receptor sequences. Part B (in orange) and D (in green) are the sequence of our exon 1 fragment and the sequence of exon 2, respectively. The vertical red line represents the position of the presumed intronic sequence.

Chimeric receptor is an approach that has been used several times in the melatonin receptor domain to pinpoint the particular role of a given segment of the receptor or to understand which part of the protein drives the melatonin binding [32,33]. A chimeric MT_2_ receptor was engineered using the sequences we were able to amplify (full exon 2 and partial exon 1) (Figure 12). The chimeric gene was based on a typical melatonin MT_2_ receptor with two exons. We deduced the unknown sequences using the consensus of six MT_2_ mammalian receptor sequences: *Marmotta marmotta* (XM_015489993), *Cricetulus griseus* (XM_007636225), *Rattus rattus* (NM_001100641), *Peromyscus maniculatus bairdii* (XM_006990545), *Microtus ochrogaster* (XM_005347416), and *Mus musculus* (AB377226). The comparison of our exon 1 sequence with that of *Peromyscus maniculatus bairdii* indicated there was an extra base located at position 77. To conserve the reading frame, we deleted this extra base in our sequence (Figure 11).

#### 4.9.2. Establishment of the Transient COS7-Chimeric MT_2_ Cell Line

The coding sequence for the *Cricetus cricetus* chimeric melatonin receptor was synthesized by Thermo Fisher Scientific (Waltham, MA, USA) and subcloned into the pCI-neo expression vector (Promega, Madison, WI, USA). The COS7 cells were obtained from the American Type Culture Collection and were maintained in DMEM GlutaMAX (Thermo Fisher Scientific) supplemented with 10% (*v*/*v*) fetal calf serum. Nucleofection of COS7 cells was performed according to the manufacturer’s instructions using the Nucleofector™ machine (Amaxa, Cologne, Germany). Adherent COS7 cells were washed in phosphate-buffered saline (PBS), trypsinized, and gently resuspended in Nucleofector™ solution V to a final concentration of 7 × 10^6^ cells/100 μL. Next, 5 μg of pCI-neo/MT_2_ vector was mixed with 100 µL of the COS7 cell suspension, transferred to a 2.0 mL electroporation cuvette, and nucleofected using program W-001 and an Amaxa Nucleofector™ apparatus. Just after the nucleofection, 500 µL of media supplemented with 20% (*v*/*v*) fetal calf serum was added to the cell suspension and cultured in a humidified 37 °C, 5% CO_2_ incubator for 20 min. Fourteen million cells were transferred to a T225 flask containing 50 mL of media supplemented with 20% (*v*/*v*) fetal calf serum and cultured in a humidified 37 °C, 5% CO_2_ incubator. Two days after nucleofection, the cells were harvested in PBS, pelleted, and stored at −80 °C until use.

### 4.10. Whole Cell and Membrane 2-[^125^I]-Iodomelatonin Binding Assay

We used these assays exactly as described previously [25]. In brief, membranes were incubated for 2 h at 37 °C in a final volume of 250 µL of a binding buffer containing 50 pM 2-[^125^I]-iodomelatonin for competition experiments. Non-specific binding was determined using 10 µM melatonin. The reaction was stopped by rapid filtration through GF/B Unifilters, followed by three successive washes with ice-cold buffer. For saturation assays, the density of binding sites (Bmax) and the dissociation constant of the radioligand (KD) were calculated according to the Scatchard method. Data were analyzed using PRISM (GraphPad Software Inc., San Diego, CA, USA). A similar protocol was used when the binding was performed on whole cells, as described by Legros et al. [34].

## Figures and Tables

**Table 1:** Various sources of melatonin receptors cloned and deposited by our laboratory.

Accession Number	Animal Name	Protein Names
MG598322	European hamster (*Cricetus cricetus*)	Melatonin receptor type 1A (MT_1_) mRNA, complete cds
MG598323	European hamster (*Cricetus cricetus*)	Melatonin receptor type 1B (MT_2_) mRNA, partial cds
MG784343	Platypus (*Ornithorhynchus anatinus*)	Melatonin receptor type 1C (Mel1c) mRNA, complete cds
KF774288	Short-nosed fruit bat (*Cynopterus titthaecheilus*)	Melatonin receptor type 1B (MT_2_), mRNA, complete cds
KC238664	Free-tailed bat (*Tadarida brasiliensis*)	Melatonin receptor type 1B (MT_2_), mRNA, complete cds
KF486508.1	Sparrow hawk (*Accipiter nisus*)	Melatonin receptor type 1B variant (MT_2_), mRNA, complete cds
KF486510	Imperial eagle (*Aquila heliacal*)	Melatonin receptor type 1B variant (MT_2_) mRNA, complete cds
KF486512.1	King penguin (*Aptenodytes patagonicus*)	Melatonin receptor type 1B variant (MT_2_) mRNA, complete cds
KF486516.1	Humboldt penguin (*Spheniscus humboldti*)	Melatonin receptor type 1B variant (MT_2_) mRNA, complete cds
KF486517	Ural owl (*Strix uralensis*)	Melatonin receptor type 1B variant (MT_2_) mRNA, complete cds
KF486518	Clawed frog (*Xenopus laevis*)	Melatonin receptor type 1C (Mel1c) mRNA, complete cds
EU679365.1	Common sheep (*Ovis aries*)	Melatonin receptor type 1B (MT_2_) mRNA, complete cds

**Table 2:** Binding affinities of reference ligands at MT_1_ receptors from *Cricetus cricetus*. Comparison with the human MT_1_ and MT_2_ receptors.

	Hamster MT_1_	Human MT_1_	Human MT_2_
MLT	8.6	9.6	9.3
2I-MLT	9.6	10.7	9.8
6 Chloro MLT	8.4	8.7	9.6
Luzindole	5.5	6.6	7.6
4PPDOT	5.9	6.8	8.9
S 20098	9.1	9.9	9.9
FLN68/ramelteon	9.6	10.1	10.3
D600 (+/−)	5.9	7	<5
S20928	5.4	7.1	7
S21278	4.4	6.2	6.2
S22153	7.2	8.2	8
S70254	5.9	7	9
S73893	7.5	8.4	8.1
S75436	8.0	7.9	8.9
S27128	8.7	8.9	9.2
Div 880	5.9	6.1	8
SD6	9.4	9.9	9.9
SD1881	8.2	8.8	8.6
SD1882	6.8	7.8	7.9
SD1918	6.8	7.3	7.3

The chemical names of the compounds are as follows: MLT: melatonin; 2I-MLT; 2-idomelatonin; 6 Chloro MLT: 6-chloromelatonin; Luzindole: *N*-acetyl-2-benzyltryptamine; 4P-PDOT: *N*-[(2*S*,4*S*)-4-phenyl-1,2,3,4-tetrahydronaphthalen-2-yl]propanamide; D600, methoxyverapamil; S20928, (*N*-[2-(1-naphthyl)ethyl]cyclobutanecarboxamide); S21278, *N*-[2-(6-methoxy benzimidazol-1-yl)ethyl] acetamide; S22153, *N*-[2-(5-ethylbenzothiophen-3-yl)ethyl]acetamide; S70254: 2-iodo-*N*-2-[5-methoxy-2-(naphthalen-1-yl)-1H-pyrrolo[3,2-b]pyridine-3-yl])acetamide; S73893, *N*-[3-methoxy-2-(7-methoxy-1-naphthyl)propyl]acetamide; S75436, 2-fluoro -*N*-[3-hydroxy-2-(7-methoxy-1-naphthyl) propyl] acetamide; S27128-1, *N*-[(8-methoxy -6-nitro-1H-indol-3-yl) ethyl]acetamide; DIV880: 2-(2-[(2-iodo-4,5-dimethoxy-phenyl) methyl] -4,5-dimethoxyphenyl; SD6: *N*-[2-(5-methoxy-1Hindol-3-yl)ethyl] iodoacetamide; SD1881 (6-iodomelatonin): *N*-[2-(6-iodo-5-methoxy-1H-indol-3-yl) ethyl]acetamideiodomelatonin; SD1882 (4-iodomelatonin): *N*-[2-(4-iodo-5-methoxy-1H-indol-3-yl) ethyl]acetamide and SD1918 (7-iodomelatonin): *N*-[2-(7-iodo-5-methoxy-1H-indol-3-yl) ethyl]acetamide. Binding experiments were performed with 2-[^125^I]-iodomelatonin as a radioligand, Experiments were run in triplicate, using at least two different batches of membranes from stably transfected CHO cells. Concentration isotherms were obtained using 10 concentrations of each products from 10^−13^ to 10^−4^ M.

**Table 3:** List of primers used for the attempts to clone *Cricetus cricetus* MT_1_ and MT_2_ genes.

**A. Primers used for the amplification of the *Cricetus cricetus* MT_1_ gene**			
**Forward Primers**		**Reverse Primers**	
TGCGCTGCGGTGAGACACCCAGGGGACC		GCGTTCCTGAGCTTCTTGTTGC	
ATGAAGGGCAATGGTAGCACTCTGCTCAATGCC		GCGTTCCTGAGCTTCTTGTTGC	
ATGAAGGGCAATGGTAGCACTCTGCTCAATGCC		CCGTATATAATTGCATTGAGGCAGCTG	
CCGCTACTGCTACATTTGCCACAGTCTC		CCGTATATAATTGCATTGAGGCAGCTG	
CAGGAAATATATTTGTGGTGAG		TTAAACAGAGTCCACCTTTA	
GTACTTTTTGCTATTTGCTGGGC		AAGACCCCAACCAGTGTGGATAATC	
**B. Primers used to amplify exon 1 of *Cricetus cricetus* MT_2_**			
**Forward Primers**		**Reverse Primers**	
ATGCCTGAGAACAGTTCTGTGCCAATTGCTGCG (F0)		CGCAGCTTGCGGTTCCTGAGCAC	
		CGCGTTCCGCAGCTTGCGGTTCC	
GCTGCGAGGCTGGTGGGCTGGCAGTGCGCT (F3)		CGCAGCTTGCGGTTCCTGAGCAC	
		CGCGTTCCGCAGCTTGCGGTTCC	
Note: The reverse primers are located in exon 1			
**C. Primers designed based on the CHO melatonin receptor 2 sequence to amplify exon 1 of the *Cricetus cricetus* MT_2_ gene**			
**Forward Primers**		**Reverse primer**	
ACCCCCACAGTTGCAAACAGAATGCACATCCG		ATTAGAGGATCCTCTTGTACTCCCTGCGGAAGTTCTGGTTC	
CAGAATGCACATCCGATGCCCTGAGAACA			
TCCGATGCCCTGAGAACAGCTCTGT			
ATGCCTGAGAACAGCTCTGTCTCCAATTG			
**D**. **Primers sequences used for exon 1 amplification using genomic DNA as the template**			
**Forward Sequences**	**Reverse Sequences**		**Size of Amplicons**
GCCTCTTCCTAGCACTTCGCTAG	GTGGGATGTGAAAGGATCTAAG		411 bp
CTGCGCGGTGAGGGGGCAGCGGG	GTGGGATGTGAAAGGATCTAAG		389 bp
GCCCCAAGCAGTACTCACCTTG	GTGGGATGTGAAAGGATCTAAG		363 bp
GCTTAGTCCCGATGCCCTGAG	GTGGGATGTGAAAGGATCTAAG		321 bp
